# Combined diaphragm and lung ultrasound profiling in healthy full-term neonates: a study of early postnatal function

**DOI:** 10.1007/s00431-026-06850-5

**Published:** 2026-03-21

**Authors:** Ioannis Koutras, Ilias Chatziioannidis, Angeliki Kontou, Abraham Pouliakis, Kosmas Sarafidis

**Affiliations:** 1 Radiology Department, Ippokrateion General Hospital, 54642 Thessaloniki, Greece; 2 1st Department of Neonatology and Intensive Care Unit, Aristotle University of Thessaloniki, Ippokrateion General Hospital, 54642 Thessaloniki, Greece; 3 2nd Department of Pathology, National and Kapodistrian University of Athens, Attikon University Hospital, 12462 Athens, Greece

**Keywords:** Diaphragm, Lung, Ultrasound, Adaptation, Neonate

## Abstract

Diaphragmatic ultrasound (DUS) and lung ultrasound (LUS) are increasingly applied to evaluate neonatal respiratory muscle performance and lung aeration, respectively. This prospective, observational, single-center study assessed DUS of both hemi-diaphragms and LUS in healthy full-term neonates on days of life (DOL) 1 and 3 to profile postnatal physiology and establish normative values. DUS metrics included diaphragmatic excursion (DE), contraction velocity (DCV), inspiratory diaphragm (DTi) and expiratory diaphragm (DTe) thickness, diaphragmatic thickening fraction (DTF), and inter-hemi-diaphragm DTF difference (ΔDTF). A six-zone, 3-point scoring system was used to evaluate LUS aeration. Twenty newborns (gestational age 39.0 ± 1.2 weeks, birth weight 3334 ± 343 g) were evaluated. No significant differences were observed in DE, DCV, DTi, DTe, DTF, or ΔDTF between DOL 1 and 3. Sex had no effect on DUS parameters. Cesarean-born neonates demonstrated significantly lower DE and DTF on DOL 1, but values were comparable by DOL 3. LUS aeration scores were similar between DOL 1 and DOL 3 (medians (Q1–Q3): 1 (0–1.3) and 1 (0–1), respectively, *p* = 0.244), with no differences by delivery mode or sex. Intra-observer reliability was excellent for nearly all DUS metrics on DOL 1 and DOL 3 (intraclass correlation coefficients > 0.90). *Conclusion*: Diaphragmatic function is stable in healthy neonates without significant pulmonary involvement on LUS during the first three postnatal days. Mode of delivery appears to transiently influence diaphragmatic performance on DOL 1. Combined DUS and LUS provide complementary insights into neonatal respiratory adaptation and may serve as reference values for clinical practice and research.

**Table:** 

**What is Known:** • *Point-of-care diaphragmatic ultrasound (DUS) and lung ultrasound (LUS) are increasingly utilized as safe, bedside tools for assessing neonatal respiratory distress and supporting clinical decision-making.*
**What is New:** • *This is the first study to evaluate both DUS and LUS together in healthy term newborns during the first 3 days of life, demonstrating stable diaphragmatic function transiently influenced by delivery mode but independent of newborn sex.*

**Supplementary Information:**

The online version contains supplementary material available at 10.1007/s00431-026-06850-5.

## Introduction

Evaluation of neonatal respiratory status has traditionally relied on clinical assessment, chest radiography, and blood gas analysis. These methods, however, have limitations [1, 2], while in the case of chest X-ray, fragile newborns are exposed to ionizing radiation [3].

In recent years, point-of-care diaphragmatic ultrasound (DUS) and lung ultrasound (LUS) have emerged as safe, bedside techniques that provide valuable insights into respiratory physiology and assist clinical decision-making. DUS allows detailed assessment of the diaphragm—the principal respiratory muscle—by measuring excursion, thickness, and thickening fraction across the respiratory cycle [4, 5]. Not surprisingly, it has been mainly applied to predict successful extubation in critically ill adults [4, 6], children [7, 8], and preterm neonates [9, 10].

By contrast, LUS evaluates lung aeration through artifacts such as A-lines, B-lines, and lung sliding. This technique is increasingly used in diagnosing common neonatal respiratory disorders including respiratory distress syndrome, transient tachypnea, meconium aspiration syndrome, and pneumothorax as well as for assessing other neonatal conditions [11, 12]. For respiratory distress syndrome in particular, LUS demonstrates higher sensitivity and specificity than chest radiography (up to 90% and 98%, respectively) [2]. In addition**,** the LUS score—more recently suggested to be referred to as the LUS aeration score [13]—has been validated for diagnosing and grading neonatal respiratory distress, with meta-analyses reporting pooled sensitivity of 0.92 and specificity of 0.95 [14]. In addition, the LUS aeration score has been used to predict failure of non-invasive respiratory support guiding surfactant therapy [15] or the need for invasive ventilation [16, 17], as well as assess risk of bronchopulmonary dysplasia development [18].

Nevertheless, despite the promising value of each modality, dual assessments remain uncommon. To the best of our knowledge, only one recent prospective study in neonates has examined the ability of combined DUS and LUS to predict extubation success in extremely preterm infants. In that study, LUS was superior to diaphragmatic indices in predicting extubation success [9]. Similarly, data in children are sparse. One investigation evaluated weaning failure in patients aged 1 month to 18 years undergoing cardiopulmonary bypass for congenital heart disease, assessing the development of diaphragmatic dysfunction due to phrenic nerve injury or increased extravascular lung water using DUS and LUS [19]. Existing evidence on the combined versus individual use of these modalities in older age groups is more encouraging. In critically ill adults, diaphragmatic dysfunction has been linked to loss of lung aeration and difficult ventilator weaning [20]. In addition, combined DUS and LUS have been used to assist in estimating pulmonary function in adults undergoing preoperative evaluation, particularly in patients unable to reliably perform conventional pulmonary function tests [21]. During the coronavirus disease 2019 pandemic, both LUS and DUS were used to assess 30-day outcomes in adult patients with suspected or confirmed disease who presented to the emergency department [22]. Notably, a recent review discusses the use of this combined ultrasound approach for the holistic evaluation of acute respiratory distress syndrome [23]. It therefore appears that combined DUS and LUS provide a non-invasive, bedside approach that can be applied across clinical settings, facilitating early detection of respiratory dysfunction and supporting timely respiratory management.

With specific regard to DUS metrics, it is worth emphasizing that existing normative data in preterm and term neonates—particularly within the first 24–48 h of life—remain limited in the current literature [24–28]. Other studies have described diaphragmatic function beyond the first postnatal week and at different time points up to early infancy [29], or from 0–6 months of age up to later childhood [30], leaving the early neonatal adaptation period underexplored.

In this context, we primarily aimed to evaluate diaphragmatic function, with complementary assessment of lung aeration serving as an indicator of lung parenchymal status early after birth, thereby providing insights into normal postnatal respiratory physiology in full-term newborns.

## Methods

### Study design and population

This prospective, observational, single-center clinical study was conducted at our center in October and November 2025. Eligibility criteria included inborn healthy full-term (≥ 37 weeks’ gestation) neonates roomed-in with their mothers in the postnatal ward of the affiliated obstetric department. Neonates who were clinically unstable after birth or had known or suspected congenital anomalies or neuromuscular diseases were excluded. Written informed consent was obtained from both parents prior to enrollment. DUS and LUS were performed on the first and third days of life (DOL) by a single experienced investigator (I.K). Lack of parental consent and unavailability of the investigator performing the ultrasound examination were additional exclusion criteria. For imaging, neonates were temporarily transferred to our Neonatal Intensive Care Unit due to hospital logistics (access to appropriate equipment), the need for standardized imaging conditions, and safety requirements (e.g., thermal stability, with infants placed in an open incubator). Ultrasound examinations were performed using a portable system (Vivid iq, GE Healthcare). Images and cine loops were acquired during quiet, rhythmic breathing, avoiding crying, yawning, sighing, or movement. To ensure this, infants were fed an hour before the examination, and no sedative drugs were used, except for an oral glucose solution when needed. Nursing staff also helped maintain the infants in a calm state during the examination. Perinatal and neonatal characteristics were recorded, including gestational age, race/ethnicity, birth weight and height, sex, mode of delivery (vaginal (VD) vs. cesarean section (CS)), and Apgar scores at 1 and 5 min after birth. The respiratory rate at the first ultrasound examination (DOL 1), as well as the postnatal age at which the DOL 1 and DOL 3 ultrasound examinations were performed, were also recorded.

### Ultrasound measurement techniques and metrics

A. Diaphragmatic ultrasound

**Fig. 1:**
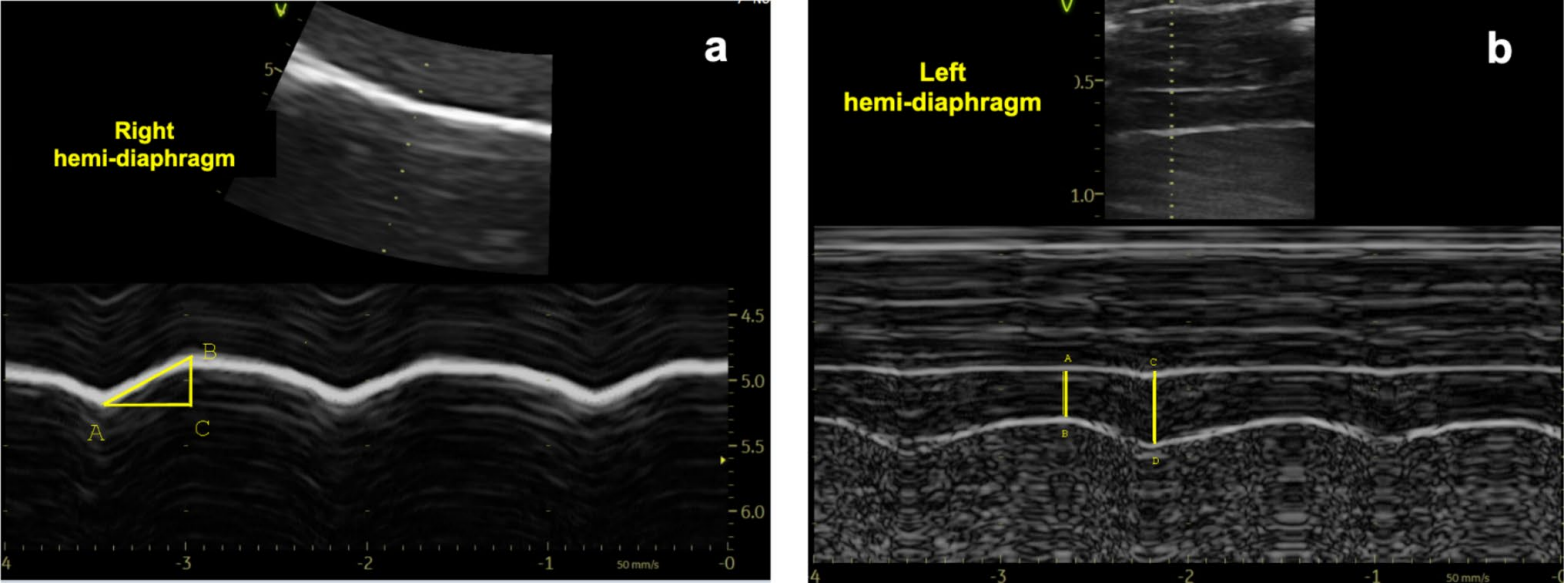
**a** Measurement of diaphragmatic excursion (DE) and inspiratory time (Ti): BC = DE, AC = Ti. **b** Measurement of diaphragm thickness: AB = DTe (expiratory diaphragm thickness), CD = DTi (inspiratory diaphragm thickness)

Diaphragmatic motion was assessed using a micro-convex probe (8 MHz) positioned subcostally at the right midclavicular line and the left anterior axillary line for the right and left hemi-diaphragm, respectively. In B-mode, the diaphragm was visualized as a hyperechoic curved line separating the lung from the liver on the right side and from the spleen or stomach on the left side. In M-mode, diaphragmatic excursion (DE) was measured as the vertical displacement between the most caudal point of the diaphragm at end-inspiration and the most cranial point at end-expiration, with the mean value of three respiratory cycles recorded [4]. Inspiratory time (Ti) was defined as the temporal interval between the caudal and cranial peaks of diaphragmatic motion in M-mode (Fig. 1a). Diaphragm contraction velocity (DCV) was calculated as DE/Ti [26].

Diaphragm thickness was assessed using a high-frequency linear “hockey stick” probe (8–18 MHz) in the zone of apposition (ZOA), between the 8th and 10th intercostal spaces, along the anterior to midaxillary lines bilaterally. In B-mode, the diaphragm was visualized as a hypoechoic band bordered by two echogenic lines: the superficial pleural membrane and the deeper peritoneal membrane. In M-mode, inspiratory diaphragm thickness (DTi) was defined as the maximal perpendicular distance between the pleural and peritoneal lines, and expiratory diaphragm thickness (DTe) as the minimal distance (Fig. 1b). Measurements were averaged over at least three respiratory cycles. The diaphragmatic thickening fraction (DTF) was calculated using the formula: DTF = (DTi-DTe)/DTe × 100% [9]. The difference in DTF (ΔDTF = DTF right − DTF left) between the two hemi-diaphragms was also calculated in the present study.

B. Lung ultrasound

LUS was performed using the same linear probe (8–18 MHz). Three chest zones were examined on each hemithorax (upper anterior, lower anterior, and lateral). Each lung zone was interrogated intercostally with the probe positioned perpendicular to the ribs and costal cartilages, and the imaging depth set at 3 cm. LUS patterns were scored as follows: A-lines were assigned a score of 0, the presence of ≥ 3 non-coalescent B-lines was scored as 1, coalescent B-lines, with or without small subpleural consolidations < 1 cm or < 0.5 cm/kg in depth, were scored as 2, and extensive consolidations were scored as 3. The cumulative score from the six lung zones constituted the total LUS aeration score (range: 0–18) [13].

### Outcomes

#### Primary outcome

To provide diaphragm metrics (of both hemi-diaphragms) in healthy term neonates on DOL 1 and DOL 3.

#### Secondary outcome

To complementarily assess lung aeration as an indicator of lung parenchymal status early after birth.

#### Exploratory outcomes

To explore DUS metrics in relation to measurement side (right vs. left), DOL, sex, and mode of delivery.

### Statistical analysis

The statistical analysis was performed using R version 4.5.1. Descriptive characteristics for quantitative data were expressed as median and quartile 1 (Q1) to quartile 3 (Q3), range, or mean ± standard deviation, depending on normality. Statistical tests included independent samples *t*-test or Mann–Whitney *U* test (the latter when normality was not confirmed via the Shapiro–Wilk test), and for dependent data (DOL 1 vs. DOL 3), paired *t*-test or Wilcoxon signed-rank test. Correlations between arithmetic data were evaluated using Spearman’s correlation coefficient (*r*_s_). The significance level (*p*-value) was set at 0.05, and two-sided tests were applied when appropriate. Intra-observer reliability was evaluated by repeated offline measurements on stored DUS images after a washout period, with the observer blinded to the prior results. Reliability was quantified using the intraclass correlation coefficient (ICC; two-way mixed-effects model, absolute agreement), applied via the *irr* package (version 0.84).

## Results

**Fig. 2:**
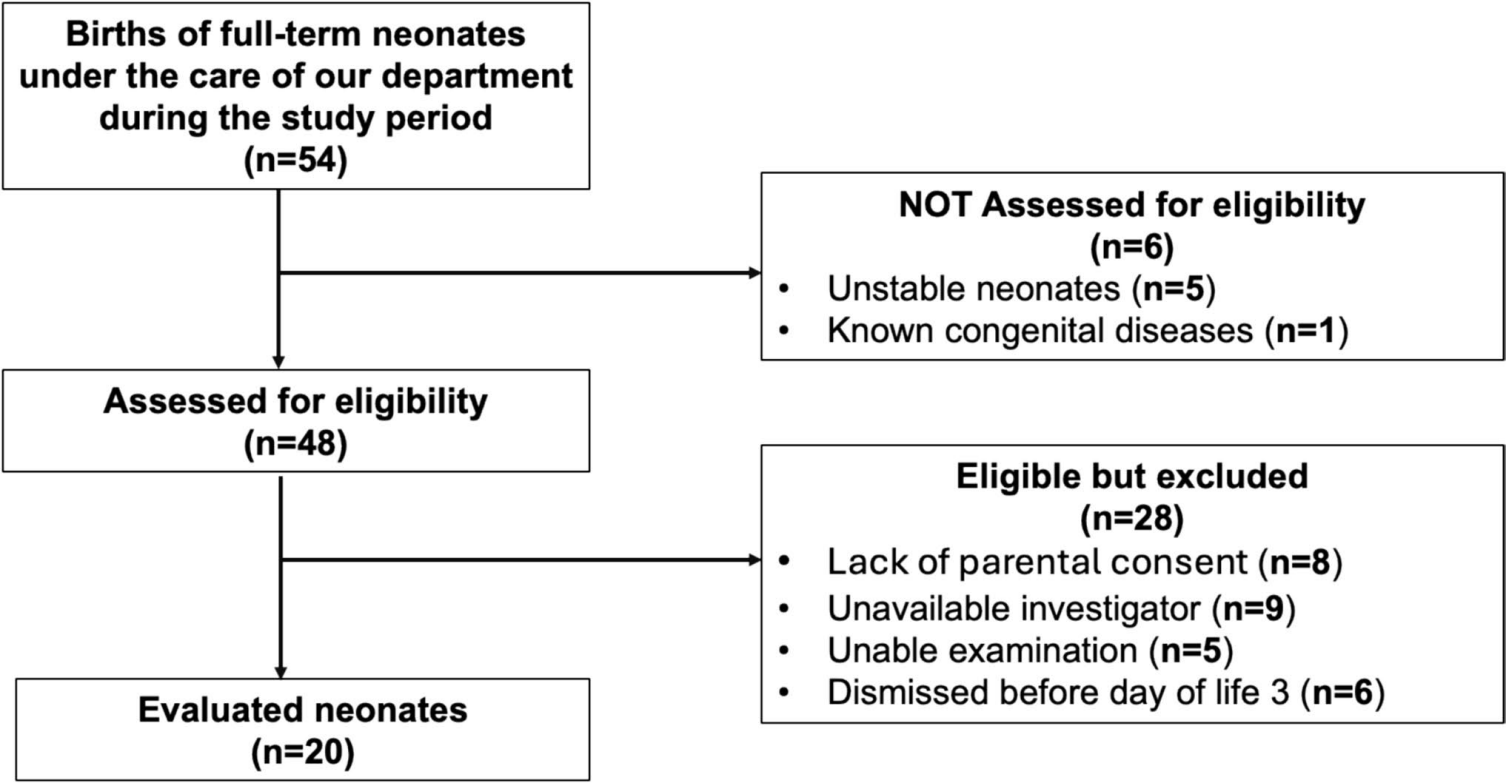
Study flow diagram

Twenty healthy, appropriate for gestational age full-term neonates (10 male/10 female), infants were evaluated, as shown in the study flow diagram (Fig. 2). All neonates were of Caucasian race, with 75% of them being of Greek ethnicity, 15% Albanian, and 10% Georgian. The mean gestational age was 39 ± 1.2 weeks, the mean birth weight was 3335 ± 334 g, and the mean length was 51 ± 1.9 cm. Half were born via VD, half via CS. Of the CSs, eight were elective (maternal request (*n* = 2), intrahepatic cholestasis of pregnancy (*n* = 2), maternal hypertension (*n* = 2), and previous CS (*n* = 2)), and two were emergency procedures (failure to progress in labor (*n* = 1) and fetal bradycardia (*n* = 1)). No studied neonate required resuscitation at birth and only routine care was applied. Apgar scores were 8 ± 0.3 and 9 ± 0 at 1 and 5 min after delivery, respectively. All infants had a normal respiratory rate during the first clinical examination performed after birth (mean 52.2 ± 4.4 breaths per minute). Ultrasound assessments were performed at a median of 17 (10–26.2) h after birth on day of life (DOL) 1 and 66.5 (58.3–72.7) h after birth on DOL 3.

Diaphragmatic function metrics on DOL 1 and 3 are presented in Table 1. DE, DCV, DTi, DTe, DTF, and ΔDTF remained stable. Sex had no effect on diaphragmatic function at both time points of the study (Table S1). However, cesarean section-born neonates showed significantly lower DE (both sides) and DTF (left side) on DOL 1 (Table 2).

**Table 1:** Diaphragmatic function metrics on days of life (DOL) 1 and 3

	DOL 1			DOL 3			DOL 1 vs. DOL 3	
	Right hemi-diaphragm	Left hemi-diaphragm	P1 Right vs. Left hemi-diaphragm	Right hemi-diaphragm	Left hemi-diaphragm	P2 Right vs. left hemi-diaphragm	P3 Right hemi-diaphragm	P4 Left hemi-diaphragm
DE (mm)	4.07 (3.38, 4.93)	4.70 (3.47, 5.26)	0.130*	4.21 (3.58, 5.04)	4.61 (3.54, 5.33)	0.546*	0.550*	0.571*
DCV (mm/sec)	9.89 (8.08, 13.00)	11.51 (9.31, 13.19)	0.090*	10.18 (9.39, 15.26)	12.44 (8.83, 16.69)	0.165*	0.784*	0.648*
DTi (mm)	2.34 ± 0.46	2.12 ± 0.56	0.124	2.35 ± 0.47	2.12 ± 0.63	0.038	0.921	0.958
DTe (mm)	1.80 ± 0.34	1.67 ± 0.49	0.233	1.80 ± 0.40	1.59 ± 0.49	0.062	0.974	0.609
DTF (%)	30.3 ± 13.5	29.0 ± 11.1	0.710	31.7 ± 13.5	33.8 ± 11.8	0.518	0.709	0.218
ΔDTF (%)	1.4 ± 16.1		Not applicable	− 2.0 ± 13.9		Not applicable	0.506	

All comparisons between paired time points were performed using paired *t*-tests, except where data violated normality assumptions—in those cases (marked with *), the Wilcoxon signed-rank test was applied and results are presented as median (Q1, Q3)

**Table 2:** Diaphragmatic function metrics on days of life (DOL) 1 and 3 according to mode of delivery

Time point	Side	Metrics	Vaginal delivery (*n* = 10)	Cesarean section (*n* = 10)	*p*-value *
DOL 1	Right hemi-diaphragm	DE (mm)	4.55 (3.94, 5.08)	3.54 (3.09, 4.14)	0.041
		DCV (mm/sec)	9.89 (9.14, 11.11)	9.74 (7.45, 13.23)	0.650
		DTi (mm)	2.49 ± 0.57	2.18 ± 0.27	0.138
		DTe (mm)	1.90 ± 0.38	1.70 ± 0.27	0.183
		DTF (%)	30.96 ± 12.98	29.69 ± 14.67	0.840
	Left hemi-diaphragm	DE (mm)	5.31 (4.79, 6.46)	3.62 (2.93, 4.48)	0.005
		DCV (mm/sec)	11.92 (10.69, 17.71)	9.57 (7.83, 12.57)	
		DTi (mm)	2.04 ± 0.46	2.21 ± 0.66	0.545
		DTe (mm)	1.50 ± 0.31	1.83 ± 0.59	0.241
		DTF (%)	36.42 ± 10.19	21.50 ± 5.89	0.001
	ΔDTF (%)		− 5.467 ± 15.76	8.19 ± 14.01	0.056
DOL 3	Right hemi-diaphragm	DE (mm)	4.38 (4.02, 5.27)	3.65 (3.33, 4.35)	0.140
		DCV (mm/sec)	12.08 (9.85, 21.49)	9.87 (8.92, 11.44)	0.290
		DTi (mm)	2.37 ± 0.58	2.33 ± 0.35	0.855
		DTe (mm)	1.78 ± 0.50	1.81 ± 0.29	0.884
		DTF (%)	33.67 ± 12.03	29.77 ± 15.24	0.534
	Left hemi-diaphragm	DE (mm)	5.01 (3.91, 5.78)	3.95 (3.20, 4.64)	0.395
		DCV (mm/sec)	12.44 (9.62, 15.34)	12.73 (9.02, 17.82)	0.082
		DTI (mm)	2.36 ± 0.75	1.87 ± 0.37	0.064
		DTe (mm)	1.77 ± 0.56	1.41 ± 0.34	0.064
		DTF (%)	33.34 ± 12.16	34.18 ± 12.00	0.877
	ΔDTF (%)		0.33 ± 15.31	− 4.42 ± 12.63	0.460

An independent samples *t*-test or Mann–Whitney *U* test was used depending on normality. Results are reported as mean ± standard deviation for normally distributed data, or as median and (Q1, Q3) for non-normally distributed data

No significant difference was observed in LUS aeration score between DOL 1 and DOL 3 (medians (Q1–Q3): 1 (0–1.3) and 1 (0–1), respectively, *p* = 0.244). As shown in Table S2, normal lung patterns predominated across all regions and time points, with only minor increases in mild findings, particularly in the right lower anterior zone and the left lateral zone. Additional analysis showed comparable LUS aeration score between neonates born via VD and CS on DOL 1 (medians (Q1–Q3): 1 (0.25–1) and 1 (0.25–1.75), respectively, *p* = 0.657) and on DOL 3 (1 (0–1) for both groups; *p* = 0.617). No differences were also observed between female and male infants at either time point (DOL 1: females vs. males, 1 (0.25–1) vs. 1 (0.25–2), respectively, *p* = 0.615, DOL 3: 1 (0–1) for both, *p* = 0.615).

On DOL 1, DE showed a significant moderate positive correlation with both DTF (r_s_ = 0.58, *p* = 0.008) and DTi − DTe (*r*_s_ = 0.56, *p* = 0.01) for the left hemi-diaphragm, whereas no significant correlations were observed on the right side. On DOL 3, DE remained significantly correlated with DTF on the left (*r*_s_ = 0.49, *p* = 0.027), while no other associations reached statistical significance (Table S3).

Overall, intra-observer reliability was excellent for nearly all DUS-derived metrics on both DOL 1 and DOL 3 (ICC > 0.90). On DOL 1, DTF (right side) demonstrated good reliability (ICC = 0.89), while on DOL 3, DCV (right side) (ICC = 0.69) and DTF (left side) (ICC = 0.72) demonstrated moderate reliability and DTF (right side) demonstrated good reliability (ICC = 0.88) (Table S4).

## Discussion

This prospective, observational, single-center study primarily aimed to evaluate diaphragmatic function and secondarily lung aeration using ultrasonography on DOL 1 and 3. We found that diaphragmatic function, as indicated by specific metrics (DE, DCV, DTi, DTe, and DTF), remains stable in healthy neonates without significant pulmonary involvement documented by LUS. Sex had no effect on diaphragmatic function. However, neonates born by CS showed significantly lower DE and DTF on DOL 1, with no differences observed on DOL 3, despite both vaginally delivered and cesarean-born neonates exhibiting similarly low LUS aeration scores at both time points.

Few studies have reported DE values in term neonates, and our findings appear to be more consistent with some of these. One such study by Laing et al. (1987) was the first to report DE values (4.6 ± 0.2 mm) for the right hemi-diaphragm in healthy newborn infants [31]. Similarly, Yeung et al. reported comparable DE values in term or near term infants (4.4 ± 1.6 mm), which were significantly lower than those observed in preterm infants with BPD (6.0 ± 1.7 mm) [32]. Recently, Carvalho et al. examined 100 term neonates (24–28 h), providing reference values for DE (2.47 ± 0.72 mm) [28]. Another cross-sectional study by Martins et al. evaluated ultrasound measures of peripheral muscles and diaphragm function in 120 stable preterm and full-term infants. Regarding the diaphragm, significant differences in both thickness and excursion were observed between term infants (DE 4.6 ± 3 mm) and very preterm infants. Notably, this is one of the few investigations in the neonatal age to report on DCV, a dynamic ultrasound metric that reflects the speed of diaphragmatic contraction [26]. In adults, DCV has been studied as a predictor of successful weaning from mechanical ventilation, though results have been conflicting [33, 34]. In neonates, reduced DCV has been reported in pathological conditions such as patent ductus arteriosus [35]. Findings from the present study suggest that DCV appears to be a robust and consistent parameter in the early neonatal period, with minimal influence from perinatal factors such as mode of delivery or sex. Furthermore, our values are close to those reported by Martins et al. for term infants (1.36 ± 0.39 cm/s) [26], thereby supporting the notion that diaphragmatic ultrasound may serve as a reliable tool for the assessment of respiratory muscle function in neonatal care. A comparative table summarizing the main characteristics of studies on diaphragmatic function (including DE and DVC) in neonates and early infancy is provided as supplementary material (Table S5).

Understanding normal diaphragm thickness in healthy term neonates is essential for interpreting respiratory muscle development and function in early life. Rehan et al. examined 16 healthy term infants within two days of birth. Right hemi-diaphragm thickness was measured at inspiration and expiration during quiet sleep in prone and supine positions. Thickness was significantly greater in the prone position, while the following values were reported in the supine position: DTi (2.36 ± 0.3 mm) and DTe (1.97 ± 0.3 mm) [24]. Alonso-Ojembarrena et al. measured 33 preterm and 33 term newborns within 48 h, reporting lower DTi and DTe in preterm ones but similar DTF between the two groups [25]. Moreover, Buonsenso et al. defined normal values in 22 healthy infants aged 7–15 days [29]. The study by Carvalho et al. also provided reference values for DTi, DTe, and DTF in term neonates [28], while another study by Duyndam et al. involving 137 children (0–8 years) reported these values separately for infants aged 0–6 months [30]. Diaphragm thickness in our study aligned with literature (Table S5) and was unaffected by sex or delivery mode whereas thickening fraction remained stable.

Right-sided ultrasound is generally preferred across all ages as the liver provides a reliable acoustic window, whereas imaging the left hemi-diaphragm is more challenging due to the smaller spleen window and potential gastric air interference [4, 36]. Unlike most previous studies assessing only the right neonatal hemi-diaphragm (Table S5), we evaluated both sides. To our knowledge, only one prior neonatal study adopted a similar approach, though it compared preterm and term infants without analyzing side-to-side variation within groups [25]. In our cohort at DOL 3, right-sided DT at inspiration and expiration were slightly higher than left-sided values, although only DTi reached statistical significance (Table 1). These small lateral differences likely reflect normal anatomical variation. For both hemi-diaphragms, no significant changes in DT or DTF occurred between DOL 1 and DOL 3, indicating stable diaphragmatic structure and function in healthy term infants during the first 72 h.

Additionally, we calculated the side-to-side diaphragmatic thickening fraction difference (ΔDTF, %) as an objective, reproducible measure of inter-hemi-diaphragmatic performance [37]. We observed a minimal mean ΔDTF (suggesting a trend for symmetric diaphragmatic function) but marked interindividual variability. In healthy men and women, notable side-to-side differences have been reported [38]. Importantly, even small absolute differences in thickness (fractions of a millimeter) may translate into appreciable percentage differences.

In the present study, moderate correlations between DE and DTF were also observed on the left hemi-diaphragm at both DOL 1 and 3, suggesting a functional link between left-sided thickening and diaphragmatic movement. Correlations on the right side were weaker and non-significant, possibly due to greater interindividual variability or technical challenges. As reported by Bahdat et al. in ventilated preterm infants, the liver’s supporting effect during inspiration may mask subtle right-sided changes. These anatomical factors likely account for side-to-side differences and highlight the importance of assessing the left hemi-diaphragm, which may provide more reliable measurements [39]. Moreover, the absolute thickness change (DTe − DTi) showed a moderate correlation with DE only on the left side at DOL 1. These findings suggest that DTF may be a more reliable marker of diaphragmatic function in early neonatal life, which may explain why absolute thickness change is not reported as a separate measurement in relevant studies.

Based on previous studies reporting thinner diaphragm in healthy women than in men [38, 40], we also investigated potential sex-related differences in diaphragmatic function. Although this hypothesis could not be supported in our study, such differences may still emerge under specific pathological conditions. A large analysis in infants with bronchopulmonary dysplasia reported thinner diaphragms in males compared to females [41].

In our study population, vaginal and cesarean deliveries were equally represented, reflecting the high CS rates reported nationally and in our region (Northern Greece: 51.49%) [42]. Regardless of the underlying causes or broader implications of this epidemiological trend, we observed that the mode of delivery influences diaphragmatic activity. Neonates born vaginally had greater DE and left-sided DTF immediately after birth. By DOL 3, these differences were no longer significant, reflecting early postnatal respiratory adaptation within 48–72 h. These findings may be related to the mechanical and physiological stresses associated with vaginal delivery and, conversely, to the absence of labor-associated respiratory stimulation in cesarean sections. Labor and vaginal birth are known to facilitate lung fluid clearance and stimulate spontaneous respiratory efforts [43]. Nevertheless, our findings do not support the assumption of delayed lung fluid clearance, as we did not observe significant differences in LUS aeration scores between neonates born via VD and CS at either time point of the study. This highlights the added value of performing LUS in addition to DUS. Moreover, any variation arising from the mode of delivery appears to be functional rather than anatomical, as the differences concerned diaphragm motion (DE and DTF) rather than diaphragm thickness. Interestingly, in the two cases of emergency CS, DTF values on DOL 1 were markedly low (16.6% and 16.7% for the right and left hemi-diaphragm in failure to progress in labor, and 9.9% and 14.4% in fetal bradycardia). By DOL 3, DTF values increased in both cases (41.7%, 46.0%, 18.1%, and 31.8%), likely reflecting postnatal functional improvement. Moreover, it is worth noting that although the central distribution of DE and DTF values observed in the CS-born infants in our study was lower than that reported in other relevant studies (in which no distinction was made regarding the mode of delivery), some of our measurements still remain within the reported normal ranges (Table S5). In any case, we believe that the impact of the mode of delivery—particularly following elective or emergency CS—on diaphragmatic function and lung aeration warrants further investigation in future studies.

In this context, this study is the first to integrate both DUS and LUS in healthy term newborns. As already outlined in the introduction, although DUS and LUS each show clinical promise, their combined use remains uncommon, particularly in neonates and children. Evidence is more established in adults, supporting integrated assessment as a feasible, non-invasive bedside approach for comprehensive respiratory evaluation. In our cohort, as expected, we observed low LUS aeration score values, reflecting normal lung patterns across all regions and time points, with only minor increases in mild findings in right lower and left lateral zones (Table S2). These results align with well-aerated lungs after effective postnatal adaptation [44]. Simultaneously, DTF and DE values indicate strong respiratory muscle function in normal lungs.

Beyond the combined use of DUS and LUS, our study has several strengths. Inclusion of neonates on both DOL 1 and 3 provides rare early-life data, expanding limited evidence on diaphragmatic function in this age group. Bilateral assessments allowed detection of asymmetry potentially missed with right-sided measurements alone. Limitations include the small sample size and follow-up restricted to two early time points. We also acknowledge the methodological concerns regarding M-mode-derived DE. Diaphragm motion is inherently complex and three-dimensional, involving both anterior displacement and caudal descent during inspiration, which can make accurate quantification of inspiratory displacement challenging. Therefore, M-mode measurements are vulnerable to error if the ultrasound beam is not aligned perpendicularly to the diaphragm, potentially affecting the accuracy of DE values. As recently highlighted by Hoshino, this approach may not be sufficiently reliable for establishing reference values, although it remains clinically useful for relative and longitudinal assessments [45]. Lastly, all DUS measurements were performed by a single investigator documenting excellent intra-observer reliability for nearly all DUS-derived metrics. Nevertheless, while the presence of a single operator minimizes inter-observer variability and ensures methodological consistency, it precludes assessment of inter-observer reliability and may limit the generalizability of the findings. Recent neonatal studies have shown excellent intra-observer and acceptable inter-observer reproducibility for diaphragmatic shortening fraction in both term and preterm infants [25]. Another study in 2024 reported good intrarater and moderate interrater reliability for the diaphragmatic thickness and mobility, while DTF interrater reliability was poor [27]. Notably, as aforementioned, DTF is a derived ratio, so small absolute variations in thickness measurements may be magnified when expressed as percentages, likely contributing to the lower intra-observer reliability for DTF observed in our study.

Future studies should enroll larger cohorts with longer follow-up to track diaphragmatic maturation beyond the early neonatal period, ideally through multi-center collaboration. Such investigations should also focus on at-risk neonatal populations to better delineate early-life adaptations and potential long-term consequences. As the diaphragm is a single functional unit but current measurements sample limited sites, standardizing methodology is essential. Future work should compare imaging modalities, assess multiple regions (ZOA and crural), explore novel metrics such as displacement curve area, combined length–thickness measures, surface variability, and contraction velocity, and integrate automated image analysis to enable broader clinical implementation.

## Conclusion

Combined diaphragm-lung assessment using ultrasound provides valuable insights into early respiratory adaptation and may help establish reference values. In healthy full-term neonates, our study using this dual approach demonstrated stable, robust diaphragmatic function during the first three days of life, with no significant sex-related differences and only a transient effect of delivery mode, with vaginal birth associated with greater diaphragmatic activity compared to cesarean section.

## Supplementary Information

Below is the link to the electronic supplementary material.

Supplementary file1 (DOCX 36 kb)

## Data Availability

No datasets were generated or analysed during the current study.

## Abbreviations

CS: Cesarean section
DOL: Day of life
DE: Diaphragmatic excursion
DTF: Diaphragmatic thickening fraction
DUS: Diaphragmatic ultrasound
DCV: Diaphragm contraction velocity
ΔDTF: Difference in DTF
DTe: Expiratory diaphragm thickness
DTi: Inspiratory diaphragm thickness
ICC: Intraclass correlation coefficient
Ti: Inspiratory time
LUS: Lung ultrasound
VD: Vaginal delivery
ZOA: Zone of apposition
