# Supplementary material for: Combined diaphragm and lung ultrasound profiling in healthy full-term neonates: a study of early postnatal function
Source: Eur J Pediatr. 2026 Mar 21;185(4):196. doi: 10.1007/s00431-026-06850-5 (PMC13005841; doi:10.1007/s00431-026-06850-5)
Supplement: Supplementary file 1 — Supplementary file1 (DOCX 36 kb) [file 431_2026_6850_MOESM1_ESM.docx]

**Supplementary Table 1.** Diaphragmatic function metrics on Days of Life (DOL) 1 and 3 according to sex.

| **Time point** | **Side** | **Metrics** | **Male**  **(N=10)** | **Female**  **(N=10)** | **P-value*** |
| --- | --- | --- | --- | --- | --- |
| DOL 1 | Right  hemi-diaphragm | DE (mm) | 4.19 [3.46, 4.59] | 3.97 [3.40, 5.38] | 0.821 |
|  |  | DCV (mm/sec) | 9.22[8.22, 12.46] | 10.66 [7.76, 12.76] | 0.940 |
|  |  | DTi (mm) | 2.37±0.56 | 2.31±0.37 | 0.785 |
|  |  | DTe (mm) | 1.80±0.35 | 1.79±0.34 | 0.949 |
|  |  | DTF (%) | 30.97±14.15 | 29.67±13.54 | 0.836 |
|  | Left  hemi-diaphragm | DE (mm) | 4.82 [3.46, 5.31] | 4.50 [3.65, 5.21] | 0.880 |
|  |  | DCV (mm/sec) | 11.62 [9.96, 12.17] | 10.78 [8.90, 17.87] | 0.880 |
|  |  | DTi (mm) | 2.16±0.53 | 2.09±0.61 | 0.325 |
|  |  | DTe (mm) | 1.70±0.41 | 1.63±0.58 | 0.290 |
|  |  | DTF (%) | 28.33±13.15 | 29.59±9.40 | 0.808 |
|  | ΔDTF (%) | | 2.64±13.48 | 0.08±19.05 | 0.733 |
| DOL 3 | Right  hemi-diaphragm | DE (mm) | 3.75 [3.51, 4.49] | 4.23 [4.03, 5.54] | 0.257 |
|  |  | DCV (mm/sec) | 10.04 [8.52, 14.18] | 10.95 [9.83, 15.18] | 0.405 |
|  |  | DTi (mm) | 2.39±0.36 | 2.30±0.58 | 0.687 |
|  |  | DTe (mm) | 1.85±0.34 | 1.75±0.46 | 0.584 |
|  |  | DTF (%) | 30.49±12.68 | 32.94±14.88 | 0.696 |
|  | Left  hemi-diaphragm | DE (mm) | 4.70 [3.80, 5.28] | 4.10 [3.41, 5.29] | 0.816 |
|  |  | DCV (mm/sec) | 12.78 [8.51, 15.66] | 12.39 [10.19, 19.85] | 0.571 |
|  |  | DTi (mm) | 2.2±0.61 1 | 2.03±0.66 | 0.290 |
|  |  | DTe (mm) | 1.67±0.47 | 1.52±0.52 | 0.212 |
|  |  | DTF (%) | 33.05±10.60 | 34.48±13.37 | 0.795 |
|  | ΔDTF (%) | | -2.56±15.52 | -1.53±12.85 | 0.874 |

*An independent samples t-test or Mann–Whitney U test was used depending on normality. Results are reported as mean ± standard deviation for normally distributed data, or as median and [Q1, Q3] for non-normally distributed data.

**Supplementary Table 2.** Ultrasound scores by lung field on Days of Life (DOL) 1 and 3, and overall. Data are expressed as counts for each score, with percentages in parentheses.

|  | **Score** | **R1** | **R2** | **R3** | **L1** | **L2** | **L3** |
| --- | --- | --- | --- | --- | --- | --- | --- |
| **DOL 1** | **0** | 19 (95.0%) | 12 (60.0%) | 19 (95.0%) | 18 (90.0%) | 17 (85.0%) | 14 (70.0%) |
|  | **1** | 1 (5.0%) | 8 (40.0%) | 1 (5.0%) | 2 (10.0%) | 3 (15.0%) | 5 (25.0%) |
|  | **2** | 0 | 0 | 0 | 0 | 0 | 1 (5.0%) |
| **DOL 3** | **0** | 20 (100%) | 17 (85.0%) | 18 (90.0%) | 20 (100%) | 17 (85.0%) | 14 (70.0%) |
|  | **1** | 0 | 3 (15.0%) | 2 (10.0%) | 0 | 3 (15.0%) | 6 (30.0%) |
|  | **2** | 0 | 0 | 0 | 0 | 0 | 0 |
| **Total** | **0** | 39 (97.5%) | 29 (72.5%) | 37 (92.5%) | 38 (95%) | 34 (85%) | 28 (70%) |
|  | **1** | 1 (2.5%) | 11 (27.5%) | 3 (7.5%) | 2 (5%) | 6 (15%) | 11 (27.5%) |
|  | **2** | 0 | 0 | 0 | 0 | 0 | 1 (2.5%) |

R1: Right upper anterior zone, R2: Right lower anterior zone, R3: Right lateral zone

L1: Left upper anterior zone, L2: Left lower anterior zone, L3: Left lateral zone

**Supplementary Table 3**. Correlations of diaphragmatic excursion (DE) with diaphragmatic thickening fraction (DTF) and absolute thickness change (DTi–DTe) on Days of Life (DOL) 1 and 3.

| **Time point** | **Side** | **DE vs DTF** | **DE vs (DTi – DΤe)** |
| --- | --- | --- | --- |
| DOL 1 | Right  hemi-diaphragm | r_s_=0.37, p=0.104 | r_s_= 0.41, p=0.073 |
|  | Left  hemi-diaphragm | r_s_=0.58, p=0.008 | r_s_=0.56, p=0.01 |
| DOL 3 | Right  hemi-diaphragm | r_s_= 0.26, p=0.261 | r_s_= 0.33, p=0.16 |
|  | Left  hemi-diaphragm | r_s_=0.49, p=0.027 | r_s_= 0.05, p=0.835 |

Spearman correlation coefficients (r_s_)

**Supplementary Table 4.** Intra-observer reliability for the DUS metrics no on Days of Life (DOL) 1 and 3.

|  | **DOL 1** | | | **DOL 3** | | |
| --- | --- | --- | --- | --- | --- | --- |
| **Metric** | **ICC** | **CI** | **Interpretation*** | **ICC** | **CI** | **Interpretation*** |
| **DE (mm) R** | 0.93 | 0.84 - 0.97 | Excellent | 0.92 | 0.81 - 0.97 | Excellent |
| **DE (mm) L** | 0.93 | 0.80 - 0.97 | Excellent | 0.95 | 0.89 - 0.98 | Excellent |
| **DCV (mm/sec) R** | 0.99 | 0.97 - 0.99 | Excellent | 0.69 | 0.37 - 0.87 | Moderate |
| **DCV (mm/sec) L** | 0.99 | 0.98 – 1 | Excellent | 0.97 | 0.90 - 0.99 | Excellent |
| **DTi (mm) R** | 0.98 | 0.94 - 0.99 | Excellent | 0.98 | 0.96 - 0.99 | Excellent |
| **DTe (mm) R** | 0.97 | 0.90 - 0.99 | Excellent | 0.99 | 0.97 - 0.99 | Excellent |
| **DTF (%) R** | 0.89 | 0.76 - 0.96 | Good | 0.89 | 0.74 - 0.95 | Good |
| **DTi (mm) L** | 0.98 | 0.96 - 0.99 | Excellent | 0.94 | 0.85 - 0.97 | Excellent |
| **DTe (mm) L** | 0.99 | 0.98 – 1 | Excellent | 0.95 | 0.87 – 0.98 | Excellent |
| **DTF (%) L** | 0.92 | 0.81 - 0.97 | Excellent | 0.72 | 0.42 - 0.88 | Moderate |

**CI: Confidence interval; DE:** Diaphragmatic excursion; **DTF:** Diaphragmatic thickening fraction; **DCV:** Diaphragm contraction velocity; **DTe:** Expiratory diaphragm thickness; **DTi:** Inspiratory diaphragm thickness; ICC: Intraclass correlation coefficients*;* **L:** Left hemidiaphragm; **R:** Right hemidiaphragm.

* ICC values were interpreted as follows: <0.50, poor; 0.50–0.75, moderate; 0.75–0.90, good; and >0.90, excellent [1].

**References**

1. Koo TK, Li MY. A guideline of selecting and reporting intraclass correlation coefficients for reliability research. J Chiropr Med. 2016;15(2):155–163. doi:10.1016/j.jcm.2016.02.012.

**Supplementary Table 5.** Comparative table summarizing the main characteristics of the available studies on diaphragm ultrasound in neonates and infants. The results of the present study, for both the entire cohort and the cesarean section–born neonates (only on DOL 1, where differences were observed compared with those born vaginally), are also presented.

| **Author/**  **year of publication-reference/**  **country** | **Laing**  **1987 [1]**  **USA** | **Rehan 2000 [2]**  **USA** | **Alonso-Ojembarrena 2020 [3]**  **Spain** | **Yeung**  **2023 [4]**  **Egypt** | **Buonsenso 2023 [5]**  **Italy** | **Duyndam 2023 [6]**  **Netherlands** | **Martins 2024 [7]**  **Brazil** | **Nascimento 2024 [8]**  **Brazil** | **Carvahlo**  **2025 [9]**  **Brazil** | **Present study** | | |
| --- | --- | --- | --- | --- | --- | --- | --- | --- | --- | --- | --- | --- |
| **N** | 46 | 16 | 33 | 55 | 22 | 30 | 44 | 69 | 100 | 20 | | 10 (CS) |
| **Age at evaluation** | DOL 1-4 | DOL 1-2 | DOL 1 |  | DOL 7-15 | 0-6 months | DOL 4-5 | DOL 1-2 | DOL 1-2 | DOL 1 | DOL 3 | DOL 1 |
| **GA (wk)** | 40±1 | 39±0.9 | 39  [38-40] | 38.1±1.5 | 38.4  (35.7–40) |  | 37-41 | 39±1 | 39 (38–40) | 39.0±1.2 | | 38.5±1.3 |
| **DE (mm) R** | 4.6±0.2 |  |  | 4.4±1.6 |  |  | 4.6±3 | 6.6±1.9 | 2.47±0.72 | 4.07  [3.38, 4.93] | 4.21  [3.58, 5.04] | 3.54  [3.09, 4.14] |
| **DE (mm) L** |  |  |  |  |  |  |  |  |  | 4.70  [3.47, 5.26] | 4.61  [3.54, 5.33] | 3.62  [2.93, 4.48] |
| **DCV (mm/sec) R** |  |  |  |  |  |  | 13.6±3.9 |  |  | 9.89  [ 8.08, 13.00] | 10.18  [9.39,15.26] | 9.74  [7.45, 13.23] |
| **DCV (mm/sec) L** |  |  |  |  |  |  |  |  |  | 11.51  [9.31, 13.19] | 12.44  [8.83, 16.69] | 9.57  [7.83, 12.57] |
| **DTi (mm) R** |  | 2.36±0.3 | 2.6  [2.2‐3.2] |  | 2.7  [2.0-3.2] | 2.07±0.4 |  | 1.9±0.5 | 2.17±0.59 | 2.34±0.46 | 2.35±0.47 | 2.18±0.27 |
| **DTe (mm) R** |  | 1.97±0.3 | 1.9  [1.6‐2.4] | 1.5±0.4 | 1.5  [1.4-1.7] | 1.64±0.30 |  | 1.6±0.4 | 1.71±0.48 | 1.80±0.34 | 1.80±0.40 | 1.70±0.27 |
| **DTF (%) R** |  |  | 24  [15‐37] | 43.3±19.7 | 68  [41-94] | 27.1±12.5 | 39.6±13.3 | 17.83±11.23 | 28.02±9.9 | 30.3±13.5 | 31.7±13.5 | 29.7±14.7 |
| **DTi (mm) L** |  |  | 2.6  [1.9‐3.2] |  |  |  |  |  |  | 2.12±0.56 | 2.12 ±0.63 | 2.21±0.66 |
| **DTe (mm) L** |  |  | 1.9  [1.6‐2.4] |  |  |  |  |  |  | 1.67±0.49 | 1.59 ±0.49 | 1.83±0.59 |
| **DTF (%) L** |  |  | 32  [15‐40] |  |  |  |  |  |  | 29.0±11.1 | 33.8±11.8 | 21.50±5.89 |

**CS:** Cesarean section; **DOL:** Day of life; **DE:** Diaphragmatic excursion; **DTF:** Diaphragmatic thickening fraction; **DCV:** Diaphragm contraction velocity; **DTe:** Expiratory diaphragm thickness; **DTi:** Inspiratory diaphragm thickness; GA: Gestational age; ICC: Intraclass correlation coefficients*;* **L:** Left hemidiaphragm; **R:** Right hemidiaphragm.

**References**

1. Laing IA, Teele RL, Stark AR (1988) Diaphragmatic movement in newborn infants. J Pediatr 112:638–643.
2. Rehan VK, Nakashima JM, Gutman A, et al (2000) Effects of the supine and prone position on diaphragm thickness in healthy term infants. Arch Dis Child 83:234–238.
3. Alonso-Ojembarrena A, Ruiz-González E, Estepa-Pedregosa L, et al (2020) Reproducibility and reference values of diaphragmatic shortening fraction for term and premature infants. Pediatr Pulmonol 55:1963–1968. https://doi.org/10.1002/ppul.24866
4. Yeung T, Mohsen N, Ghanem M, et al (2023) Diaphragmatic Thickness and Excursion in Preterm Infants With Bronchopulmonary Dysplasia Compared With Term or Near Term Infants: A Prospective Observational Study. Chest 163:324–331.
5. Buonsenso D, Mariani F, Morello R, et al (2023) Ultrasound Imaging for Diaphragm Function in a Population of Healthy Infants: A Short Observational Report. Diagnostics (Basel) 13:1095. https://doi.org/10.3390/diagnostics13061095
6. Duyndam A, Smit J, Heunks L, et al (2023) Reference values of diaphragmatic dimensions in healthy children aged 0-8 years. Eur J Pediatr 182:2577–2589. https://doi.org/10.1007/s00431-023-04920-6
7. Martins MF, de Santa Maria NN, Dos Santos Camargo CC, et al (2024) Lower limit of normal of cross-sectional area of peripheral muscles and diaphragm measurements performed with ultrasound in full-term and preterm infants. Eur J Pediatr 183:4799–4806.
8. Nascimento MS, Leite FS, Silva PAL, et al (2024) Reliability and reference values for diaphragmatic excursion, thickness, and thickening fraction and quadriceps femoris muscle thickness in full-term newborns evaluated by ultrasound. Eur J Pediatr 183:3453–3460.
9. Carvalho MGS, Tomiyama JN, Terra MA, Lanza FC (2025) Diaphragmatic ultrasonography: reference values and reliability for thickness, thickening fraction, and excursion in neonates. Eur J Pediatr 184:709. https://doi.org/10.1007/s00431-025-06484-z
